# “Pure Fat Flap”—Perforator-based Adiposal Layer Only Flap for Lateral Ankle Reconstruction

**DOI:** 10.1055/a-2267-4205

**Published:** 2024-04-10

**Authors:** Seok Joon Lee, Jeongmok Cho, Changsik Pak, Hyunsuk Suh, Joon Pio Hong

**Affiliations:** 1 Department of Plastic and Reconstructive Surgery, Asan Medical Center, University of Ulsan College of Medicine, Seoul, Republic of Korea

**Keywords:** ankle, perforator flap, wound healing

## Abstract

Lateral ankle soft tissue defects pose challenges, especially in cases due to chronic pressure from cross-legged sitting, which usually present with a large dead space, small skin opening that often accompanies an open joint. Traditional reconstruction methods using fasciocutaneous flaps may result in donor site morbidity such as delayed wound healing or nerve injury. In this article, we present a case of diabetes-related lateral ankle defect successfully treated using adiposal layer only flap, also known as pure fat flap. The anatomy and the surgical technique of adiposal layer only flap were reviewed. These flaps preserve the subdermal plexus and deep fascia while obliterating dead space and providing a gliding surface for proper ankle movement. A perforator-based adiposal layer only flap was elevated from the peroneal artery and used to cover the defect. Flap perfusion was confirmed using indocyanine green video angiography and color duplex ultrasound. Patient had a successful recovery with minimal donor site morbidity. The technique expands the reconstructive microsurgeon's options for complex ankle coverage, ensuring optimal wound healing and functional outcomes.

## Introduction

Trauma, tumor, diabetes mellitus, and peripheral vascular disease are responsible for majority of cases that present with soft tissue defect of lateral ankle. 1 2 Defects related to diabetes mellitus especially presents a significant challenge in terms of reconstruction and healing. 3 In culture where sitting cross-legged on the floor is common, the lateral ankle wound becomes further difficult as they are usually presented with a large dead space, small skin opening that often accompanies an open joint.

The first line for reconstruction can be a simple rotation of a local flap or a propeller flap based on peroneal artery perforator. 4 These fasciocutaneous flaps are commonly utilized and have provided several advantages in lateral ankle reconstruction including reduced operative time and improved wound healing. However, the use of fasciocutaneous local flaps or propeller flaps, in which a skin paddle is harvested from a tight donor site often can lead to morbidity such as delayed wound healing or nerve injury.

To address these issues for diabetes-related lateral ankle defects where the dead space is far greater issue than the skin defect, the authors aimed for a flap that can obliterate the dead space while allowing smooth gliding for underneath structures. Theoretically, obliterating the dead space with fat can be anatomically ideal. Thus, the adiposal layer only flap or so called the pure fat flap based on a single perforator was designed. The pure fat flap will preserve the subdermal plexus of the skin allowing the donor site skin to heal without the need for any skin grafts, preserve the deep fascia reducing the chance of muscle hernia or nerve injury. To our knowledge, this is the first case of utilizing a pure fat flap of moderate size which was performed to obliterate the dead space and provide coverage for the lateral ankle defect.

## Case

**Fig. 1:**
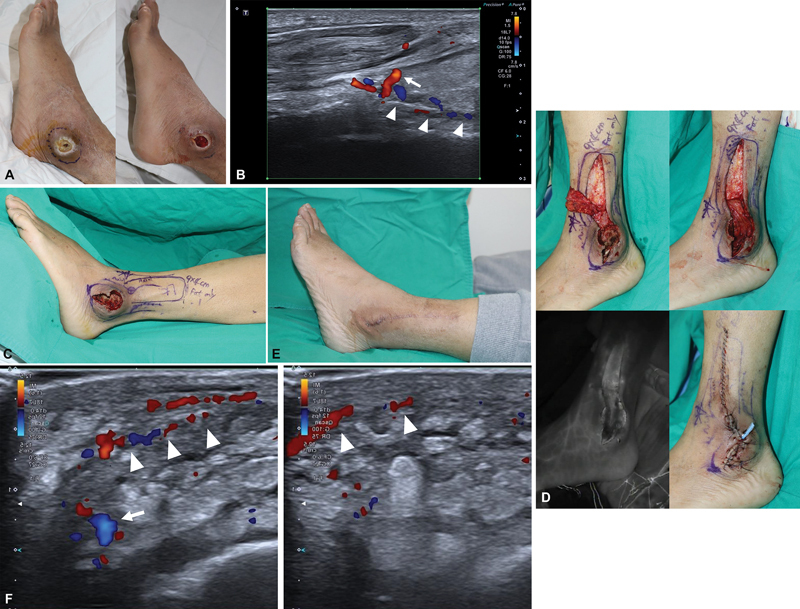
( **A** ) (Left) Preoperative photo of patient 1. (Right) Postoperative photo of patient 1 after debridement. ( **B** ) Ultrasonographic finding of patient 1, showing peroneal artery (shown by arrowheads) and accompanying perforator from peroneal artery (shown by arrow). It was traced before surgery using color duplex ultrasound. ( **C** ) Adiposal layer only flap design of patient 1 according to perforator course. ( **D** ) (Above, Left) After adiposal layer only flap elevation. (Above, Right) After flap rotation. (Below, Left) ICG video angiography after flap rotation. Note that flap is well perfused including distal portion after rotation. (Below, Right) Immediate postoperative photo. ( **E** ) Postoperative ultrasonographic finding showing intact pedicle. Peroneal artery perforator is seen (shown by arrow) and continued in axial pattern (shown by arrowheads). ( **F** ) Postoperative 8 months photo of patient 1. ICG, indocyanine green.

A 46-year-old male patient was noted with bursitis of the left lateral malleolus. The orthopaedic team performed multiple incisions and drainage without success and ultimately referred to reconstruction for the defect. Debridement was performed and resulted in a 2.5 × 2 cm skin defect and a 5 × 5 cm dead space pocket with small opening of the joint leaking fluid ( Fig. 1A ). A contrast-enhanced magnetic resonance imaging of the left ankle was performed, revealing no evidence of osteomyelitis. The patient had history of coronary intervention for 3-vessel disease, end-stage renal disease on hemodialysis, and had diabetes for 20 years. The ankle brachial index was 1.35 on the left side (1.28 on the right side), and enhanced computed tomography lower extremity angiography revealed patency of the anterior tibial artery, posterior tibial artery, and peroneal artery, which was further confirmed by ultrasound examination. Prior to coverage, duplex ultrasonography was used preoperatively to find a perforator that would allow a perforator-based adiposal layer turnover flap ( Fig. 1B ). 5 A perforator was identified from the peroneal artery.

A 9 × 4 cm turnover pure fat flap was designed based on the perforator located 2 cm from the edge of the defect ( Fig. 1C ). Dissection was performed above the deep fascia, perforator was identified, and adiposal layer only flap was elevated preserving subdermal plexus. The sural nerve was also saved. The perforator based pure fat flap was elevated and turned over to cover lateral malleolus. The indocyanine green angiography confirmed the flap perfusion. Primary closure with quilting suture was performed at the donor site to eliminate dead space. The pure fat flap obliterated and covered the defect, and the skin was closed primarily. A silastic drain was placed at the lateral ankle with a splint to immobilize the joint ( Fig. 1D ).

Postoperative process was uneventful, and the drain was removed on day 4. The follow-up duplex ultrasound on day 5 showed good flow ( Fig. 1E ). The follow-up at 8 months showed good coverage and healing with minimal donor site morbidity ( Fig. 1F ).

## Discussion

For diabetes-related lateral ankle defects with specific features such as dead space and a relatively small skin defect, several factors need to be considered. The presence of dead space in the lateral ankle defect refers to the gap or void that may exist above the lateral malleolus, which is easily seen during debridement for wound preparation. Dead space can pose a challenge to wound healing and increase the risk of complications such as fluid accumulation and infection. It is, therefore, critical to effectively manage this dead space to promote optimal healing and functional outcomes. The dynamic nature of the ankle also requires the provision of a gliding surface within the defect reconstruction.

In addressing these specific requirements, surgeons may employ various approaches. Since lateral ankle is a watershed area between two angiosomes, it has a relatively poor blood supply compared with other areas of the body leading to potential poor secondary healing. 6 Patients with diabetes mellitus or peripheral vascular disease are prone to malleolus defect to occur and they may not be a good candidate for free tissue transfer. 7 For local flap coverage, such as reverse sural artery flap, 8 it is common for distal foot wounds to become larger due to donor site morbidity. Adipofascial flap, composed of perforator, deep fascia, and fat layer spares subdermal plexus and skin for donor and this makes donor site healing more effective. They were previously employed in head and neck reconstruction 9 and lower extremity reconstruction, 4 utilizing various methods such as the adipofascial fold-down flap 10 or the venoadipofascial pedicled fasciocutaneous flap. 11 However, with deep fascia harvested together, it has possibility of muscle hernia and injuring the nerve leading to persistent pain, altered sensation, or numbness in the donor site. The pure fat flap, with proper dissection, can preserve deep fascia which poses no risk for sural nerve damage.

Perforator-based propeller flaps have become a valuable tool in reconstructive surgery, 12 allowing for the transfer of tissue from a donor site to a recipient site while preserving major blood vessels. These flaps are designed based on the concept of utilizing specific perforating blood vessels that supply the overlying tissue. With duplex ultrasound, a noninvasive imaging technique, we can identify the pedicle of interest and design and elevate flap according to pedicle course looking at actual vessels. However, we commonly encounter fat necrosis without skin necrosis after proper flap elevation according to perforator 13 14 and thus circulation to subcutaneous fat (adiposal layer) could be of question.

**Fig. 2:**
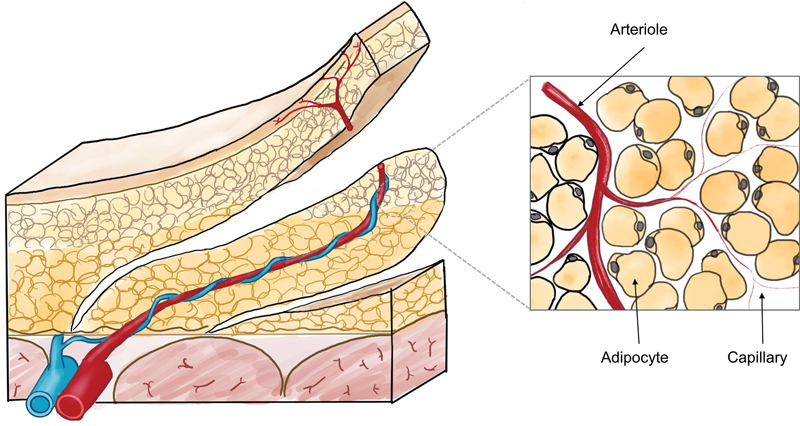
Schematic illustration of fat circulation. At subcutaneous fat level, there are no terminal capillary and cells are supplied by adjacent capillaries.

Blood circulation in the skin is indeed facilitated by capillaries, which are tiny blood vessels that form a dense network throughout the dermis, the middle layer of the skin. As for fat (adipose cell), it does not have its own circulatory system like capillaries. 15 16 Instead, the blood supply to adipose tissue comes from the surrounding capillaries. In other words, adipose tissue does not have terminal branches of blood vessels like capillaries but relies on the adjacent capillaries to maintain its metabolic functions ( Fig. 2 ). The capillaries in the skin, which are part of the overall circulatory system, serve not only the skin itself but also support the functions of underlying fat tissue. Therefore, we suggest that adiposal layer only flap should be designed more conservatively in smaller dimension than we would normally design a perforator-based propeller flap.

Gold standard of checking blood flow of adipose tissue is ^133^ Xenon washout technique, 17 however it is hard to use it in clinical setting. Alternative techniques such as Doppler ultrasound was studied, and recent study 18 comparing ^133^ Xenon washout technique and Doppler ultrasound by Lempesis et al showed ultrasound can be effectively used as alternative to check and quantify adipose tissue blood flow. Thus the circulation of adiposal layer only flap was checked with color duplex ultrasound both preoperatively and postoperatively, showing intact pedicle. Moreover, intraoperative indocyanine fluorescent angiography helps evaluate the perfusion of flaps, 19 and all flap perfusion was checked with indocyanine fluorescent angiography after elevation and rotation of the flap to the lateral ankle defect. Postoperative care is also important because stretching of flap can result in damage in pedicle such as thrombosis or ischemia. Ankle immobilization in early postoperative period will be important, and we used short leg cast for protection.

There have been previous reports of adiposal flap in finger reconstruction, which used pedicled adipose tissues based on digital artery. 20 They identified digital artery and elevated preserving pedicle, but they did not check the perfusion afterwards. With aid of indocyanine fluorescent angiography and color duplex ultrasound, we ensured the perfusion of flap. Also, digital artery-based adiposal flaps were much smaller compared with our flap being long as 7 to 9 cm.

Limitation of adiposal layer only flap exists. Further understanding of the vascularity for the adiposal layer is needed. Nevertheless, this report demonstrates the possibility and potential of using the fat only as a flap.

### Conclusion

Pure fat flap or the adiposal layer only flap may be an alternative for reconstruction in areas with large dead space, small skin defect, and joint movement. The advantages of the pure fat flap are preserving the donor site skin, allow vascular bulk tissue to obliterate dead space, and the fat to preserve good gliding function of the underlying structures. By carefully addressing the specific requirements of the defect and utilizing advanced imaging techniques, we can enhance the chances of optimal wound healing using this approach. This technique adds to the reconstructive microsurgeon's armamentarium for complex coverage of the ankle region.
